# Methodological Issues in the Neuroscience of Moral Judgement

**DOI:** 10.1111/j.1468-0017.2010.01401.x

**Published:** 2010-10-18

**Authors:** Guy Kahane, Nicholas Shackel

**Affiliations:** Oxford Uehiro Centre for Practical Ethics University of Oxford; Nicholas Shackel Department of Philosophy University of Cardiff and Oxford Uehiro Centre for Practical Ethics University of Oxford

## Abstract

Neuroscience and psychology have recently turned their attention to the study of the subpersonal underpinnings of moral judgment. In this article we critically examine an influential strand of research originating in Greene's neuroimaging studies of ‘utilitarian’ and ‘non-utilitarian’ moral judgement. We argue that given that the explananda of this research are specific personal-level states—moral judgments with certain propositional contents—its methodology has to be sensitive to criteria for ascribing states with such contents to subjects. We argue that current research has often failed to meet this constraint by failing to correctly ‘fix’ key aspects of moral judgment, criticism we support by detailed examples from the scientific literature.

In recent years scientists have turned to study the psychological and neural underpinnings of morality, and an influential strand of this research has directly drawn on the work of moral philosophers, explicitly referring to particular philosophical theories, distinctions and debates. Joshua Greene (Greene *et al.*, 2004; Greene *et al.*, 2001) and others have drawn on the longstanding philosophical dispute between utilitarian and deontological moral theories, making use of philosophical examples such as the by now famous Trolley and Footbridge dilemmas. Similar work by Borg *et al.* (2006) has looked at the deontological distinctions between act and omission and intended and foreseen consequences.

This strand of research has aimed to explain differences between types of moral judgment in neural terms. For example, it has aimed to explain why most people make certain deontological distinctions, and why some people do not. More grandly, this research has been presented as the basis for a general explanation of the opposition between utilitarian and deontological moral theories. Greene, for example, suggests that his work has shown that that the ‘controversy surrounding utilitarian moral philosophy reflects an underlying tension between competing subsystems in the brain’ (Greene *et al.*, 2004, p. 389).

In this article we will critically examine the methodology of this line of research. If this research is to succeed in explaining differences in moral judgement, its methodology needs to be appropriately sensitive to these differences. We will argue that the methodology employed by current research often fails to meet this constraint.

## 1. Conceptual Background

### 1.1 Moral Beliefs and their Ascription

The neuroscience of morality is not the science of moral *theories*, any more than the neuroscience of mathematics is the science of numbers. It is an empirical investigation concerning persons and their attributes, and moral theories—sets of normative propositions—are simply not attributes of persons. Moral beliefs and judgements *are* attributes of persons, as are acts, motivations, traits, and so forth. It is these that can be the object of empirical investigation.^1^

Moral beliefs are propositional attitudes that have as their objects moral propositions. Thus for a person to believe a given moral theory is simply for that person to believe the set of normative propositions of which that theory consists. Let us briefly consider what goes into ascribing moral beliefs to persons.

There is a special type of case where ascribing moral beliefs to a person is simple. A moral philosopher such as Peter Singer has expounded his moral beliefs in numerous books and articles. We can thus confidently ascribe to him belief in a highly determinate moral theory—a variant of act utilitarianism. Things are not so simple, however, when we ask about the moral beliefs of what we will call ‘lay moralizers'. The problem is not simply that we do not have direct evidence about their general views. Even if asked, it is doubtful that those lacking philosophical training will be able to accurately articulate many of their moral beliefs. More importantly, it is doubtful that most lay moralizers believe anything general, systematic and consistent enough to count as a moral theory. It is more likely that most believe in a messy collection of fairly specific moral rules and considerations.

So, often there is no verbal shortcut to the ascription of moral belief to lay moralizers; we need to take the longer route of collecting evidence about their verbal and nonverbal behaviour over time across a range of situations. Even when we have such evidence at hand, ascription of moral belief remains uncertain. A given act or judgement might be mandated by numerous different sets of moral beliefs, if conjoined with the right set of empirical beliefs, and the same set of general moral beliefs could lead to opposing judgements about a particular case if conjoined with different moral and empirical beliefs. The project of ascribing moral belief is made even more complicated by the fact that people are not perfectly rational and have limited capacities. It is a familiar point that due to weakness of the will and self-deception, people often fail to behave in accordance with their avowed moral beliefs. And mistaken inferences or limits of attention can create competence/performance gaps between endorsed general principles and particular judgements and acts.

### 1.2 Personal and Subpersonal

Moral beliefs are what Dennett calls personal level states, mental states *of* a person, whereas what neuroscience and much psychology study are subpersonal states and processes—information processing or neural activity taking place *in* a person's brain (Dennett, 1978). The relation between these two levels of description is controversial, and even a die-hard physicalist needn't hold that there is a simple correspondence between types of personal-level states and types of neural activity. The conceptual scheme that guides our ascription of mental states to others—what is sometimes called ‘folk psychology’—is at most a rough guide to subpersonal structure. Indeed, there may be no reflection at the personal level of important distinctions at the subpersonal one. For example, phenomenology and our conceptual scheme don't single out recognition of faces as distinct from other forms of perception, yet it is now widely believed that face recognition involves a dedicated neural module.

Why, then, should the personal level of explanation and the folk psychological practice of ascribing moral belief matter for scientific inquiry? To start with, a rough guide is still *a* guide, and it is at least likely that, in one or another way, many basic personal-level distinctions are reflected at the subpersonal level. So to ignore basic person-level distinctions, at least at the outset of inquiry, is to risk overlooking important differences at the subpersonal one. A more important reason is that the typical explananda of empirical investigation into morality (and into many other psychological phenomena) are personal-level states—in our case, common moral intuitions or patterns of moral judgement. Such research seeks to confirm or falsify causal statements such as:

Subpersonal process X causally explains why subjects tend to judge that it is morally forbidden to do act Y.

Since moral judgements are individuated at the personal level, scientific inquiry cannot help but respect the conceptual constraints governing ascription of such states to a person. If it fails to respect these constraints, then it has simply changed the subject. It may still tell us interesting things about various subpersonal processes, but it will fail to explain what is set out to explain. The change of subject can be obscured by the common failure to distinguish between these two levels of description, as when, for example, subpersonal states are described in personal-level terms.

Much research in psychology and neuroscience implicitly recognizes this point. In many areas of research, controlled laboratory settings are deployed precisely in order to enable, on the basis of limited behavioural evidence, ascription to subjects of mental states with fine-grained content. Once this person-level state has been properly ‘fixed’, the research can proceed to identify, at the subpersonal level, its correlates and causal antecedents consequences (both proximal and distal).

## 2. Three Methodological Problems

We now turn to recent neuroscientific research into moral judgment. Our discussion will largely focus on the pioneering neuroimaging studies of Joshua Greene and on research directly influenced by it. This work has shaped the methodology of much subsequent research into moral judgment, and has been widely influential both in neuroscience and in other disciplines, including moral philosophy, where it has been taken to have momentous implication for the substance and practice of normative ethics (Greene, 2008; Singer, 2005). But many of our critical points have wider application.

This strand of research sets out from a well-known philosophical dispute. In the Trolley case, a runaway trolley is about to kill five bystanders, and one can save them only by diverting it to another path, where it would kill one. The Footbridge case is similar, but here one can save the five only by pushing a stranger onto the trolley's path, again meaning that one person must die if the five are to be saved. Many philosophers recognise an intuitive moral distinction between the Trolley and Footbridge case. They believe that it's permitted to divert the trolley but not to push the stranger—a deontological distinction that utilitarians reject as spurious.^2^ Greene *et al.*'s 2001 study set out to identify the neural processes underlying judgements responding this distinction. Greene *et al.* 2004 also compared the neural processes underlying judgements that responded to this distinction with those underlying ‘utilitarian’ judgements that didn't. Greene takes his and related work to suggest a *causal explanation* of these two opposing patterns of judgement, as well as, more ambitiously, of the neural source of the traditional dispute between utilitarians such as Bentham and Mill and deontologists such as Kant.

Greene's 2001 explanandum is thus:

- The different pattern in moral judgements exhibited by a large majority of normal subjects in response to the Footbridge dilemma (and relevantly similar dilemmas) as opposed to the Trolley dilemma (and relevantly similar dilemmas).

In his 2004 study, an additional explanandum is:

(b) The statistically deviant pattern of moral judgements exhibited by the minority that chooses the utility maximising option in both the Footbridge dilemma (and relevantly similar dilemmas) and the Trolley dilemma (and relevantly similar dilemmas).

This research aims, in the first instance, to identify the subpersonal processes that correlate with these differences in moral judgment, and ultimately to explain them causally in neural terms. And on this basis, it hopes to offer a general explanation of why people believe in (and disagree about) moral theories such as utilitarianism and Kantian ethics.

What is evident, however, is that the explananda of this research explicitly refer to certain personal level states—to moral judgements with specific contents. The research is thus subject to the constraints on ascription we have outlined above. In what follows, we will draw attention to ways in which this research has failed to properly ‘fix’ the right type of personal level state. The subpersonal processes it has identified as causing or underlying these personal level states might therefore be off target, meaning that the research may fail to explain what it aimed to explain, and risks misidentifying important neurocognitive kinds.

In particular, we shall argue that this research has often failed to fix three distinct aspects of the person-level state that is the moral judgement:

1. the *type* of moral judgement made by a person (e.g. that we ought to push a stranger in front of a runaway trolley);
2. their *reason* for making that judgement (e.g. that this will save a greater number of lives);
3. the *general* moral principles or overall moral outlook that might be expressed by the judgement (e.g. that we must always maximize aggregate wellbeing).

### 2.1 Fixing the Type of Moral Judgement: Asking the Right Question

When we present subjects with, say, an image of violence, it is plausible that this will elicit a moral response. It is unclear however what moral *judgements*, if any, subjects might be making. Are they judging that it's *bad* that the victim was hurt, or that the violent person is *cruel*, or that he is behaving *wrongly*, or something else?

The studies we are concerned with do not leave things open in this way. In these studies, subjects were presented with a series of moral dilemmas, each of which describes two possible choices, making salient moral considerations for and against engaging in a certain act. Subjects are then asked to issue a moral verdict about that act. Effectively, they need to endorse a moral proposition as their response to the described scenario. The problem is that the question subjects are asked in many of these studies still leaves it unclear which moral proposition they are endorsing or rejecting.

In Greene *et al.*'s studies, and in many other studies (e.g. Ciaramelli *et al.*, 2007; Heekeren *et al.*, 2003; Moore *et al.*, 2008; Valdesolo and DeSteno, 2006), subjects were asked whether a certain act is *appropriate*. This question is ambiguous. Acts can be morally forbidden, permissible or required. Forbidden acts are neither permissible nor required, and required acts are permissible and not forbidden.^3^ Permissible acts, however, need not be required. When not required, permissible acts might simply be morally neutral (e.g. scratching one's ear) or they might be supererogatory—morally good yet beyond the call of duty.

Which of the above does ‘appropriate’ refer to? Let us set aside the problem that ‘appropriate’ could be understood to refer not to a moral property but to compliance with merely conventional rules (Borg *et al.*, 2006; Mikhail, 2008).^4^ There is a problem even if ‘appropriate’ is understood to refer to a moral property. Consider the Footbridge dilemma. Let us suppose that utilitarianism morally *requires* pushing the man (though see below). When a subject judges a given act to be appropriate, he seems to take it to be *permissible*. But, as we just saw, this means that we don't know if he also takes it to be *required*. (Indeed, some deontological views give people ‘prerogatives’ that *permit* them both to maximise wellbeing and to refuse to do so.) Worse, the fact that the subject judges an act to be permissible leaves it entirely open whether he judges the alternative as also permissible or as forbidden. To judge an act to be appropriate, then, is compatible *both* with utilitarianism and its deontological opponents.

Petrinovich *et al.*, 1993 and Koenigs *et al.*, 2007 used a different question. They asked subjects if they *would* do the act. This is worse. ‘Would’ is not a normative notion but a predictive one. It gives us information about the moral beliefs of subjects only on the assumption that, in answering the question, they believe they would behave as morality says. But often subjects would have good reason to think otherwise. Someone who is especially squeamish might predict that he won't be able to push the stranger in Footbridge, despite believing this is the right thing to do. Or someone might simply be *uncertain* as to whether he would push the stranger, even if he believes this to be morally required. Notice finally that even if subjects interpret this question in normative terms, it inherits all the ambiguities of ‘appropriate’, since it similarly doesn't distinguish the permissible and the required.

We have been drawing attention to ambiguities in the questions used by some studies. It is an empirical question whether lay subjects in fact interpret some of these questions in different ways. It may be, for example, that when subjects are asked whether they would do something, they almost invariably understand the question to be whether it is permissible.

There is, however, evidence that subjects do understand some of the above questions to mean different things, and that this difference makes a psychological difference. Borg *et al.*, 2006 asked subjects two questions: ‘Is it wrong to… ?’ and ‘Would you… ?’ They found that whereas reaction times to the first, normative question did not differ between moral and nonmoral conditions, they did differ for the second question. More importantly, if subjects think they would do something only if they also judge it not to be wrong (and vice versa), then the percentage of subjects who answered YES to ‘Is it wrong to… ?’ and YES to ‘Would you… ?’ should add up close to 100%. For example, when subjects replied to Footbridge type dilemmas, 69% judged such acts to be wrong, suggesting that 31% judged it to be permissible. Yet only 8% said they would commit such acts, though we do not know if this gap is due to squeamishness or the belief that such acts are merely permissible, not required. This finding casts doubt on the findings of previous studies that used non-normative vocabulary.

This is a fairly low-level methodological flaw, but it means that in many studies we do not even know what type of moral judgement subjects are making. Of course a battery of scenarios used in an experiment cannot be reasonably expected to rule out all possible interpretations, and normative notions are not understood in exactly the same way by everyone, let alone as philosophers understand them. But we should at least phrase our question in appropriate normative vocabulary, as was done is several other studies. In Kohlberg's classic studies of moral development (Kohlberg, 1981), subjects were asked whether they *should* do some act. Wheatley and Haidt (2005) asked subjects whether an act is *morally wrong*. And Hauser *et al.*, 2007 asked whether some act is *permissible*, and in answering, subjects could rank the act on a scale ranging from forbidden through permissible to obligatory. And for reasons highlighted above, it might be advisable to ask subjects for their responses to *both* presented options.

### 2.2 Fixing the Reasons for Judgement: Using the Right Dilemmas

Even if we knew that subjects are judging some act to be morally forbidden, this would tell us very little if we don't know *why* they did so—what moral reason governed their choice. In empirical research we are not interested in particular instances of moral judgement but in repeated patterns of judgement, whether within a subject or, more typically, across subjects. And this means that having fixed the type of judgement made, we typically need this judgement to be made for broadly the *same reasons*. To control for subjects' reasons, we need to control the content of the dilemmas we present them. In particular, if our aim is to explain the Trolley/Footbridge distinction, the content of the dilemmas posed needs to consistently involve a choice between the impersonal maximization of welfare and some deontological constraint on permissible harm.

#### 2.2.1 Dilemmas with the Wrong Content

In his studies, Greene used a battery of sixty dilemmas. These dilemmas were divided into ‘moral’ and ‘non-moral’ categories on the basis of the responses of pilot participants. The category of ‘moral’ dilemmas was further divided into what Greene calls ‘personal’ and ‘impersonal’ moral dilemmas.^5^ On Greene's definition, for a moral violation to be considered personal, it had to meet three criteria:

First, the violation must be likely to cause serious bodily harm. Second, this harm must befall a particular person or set of persons. Third, the harm must not result from the deflection of an existing threat onto a different party (Greene *et al.*, 2004, p. 389).

First used in Greene *et al.*, 2001, this battery of dilemmas (or various subsets of it) has been employed in several further studies by Greene, where he has claimed to find, for example, an association between deontological judgements and emotion and utilitarian judgement and cognition. It has also been used in related studies looking at the influence of brain damage on moral judgement, such as the studies by Koenigs *et al.*, 2007 and Ciaramelli *et al.*, 2007 of patients with damage to the ventromedial prefrontal cortex (VMPC) that claimed to find that such patients have an ‘an abnormally “utilitarian” pattern of judgements' (Koenigs *et al.*, 2007, p.1).

We shall later discuss the ways these studies fall short of providing a basis for assertions about ‘utilitarian’ judgement or about an ‘abnormal utilitarian tendency’. For the moment we only wish to consider whether this battery is even adequate as a means for investigating the deontological distinction between Trolley and Footbridge. First we need to point out a common mistake about Greene's personal/impersonal distinction.

The Trolley dilemma is an impersonal dilemma and the Footbridge one a personal only because of the deflection criterion. Unfortunately, and perhaps because that criterion is so closely tuned to the Trolley case, this has led some researchers to refer to the Personal/Impersonal distinction as if it is a synonym for the Trolley/Footbridge distinction. This is mistaken. The distinction between personal and impersonal dilemmas is not itself the explanandum of the study, but is rather an explanans. It is an initial *hypothesis* about the psychological factors that result in different judgements across the two types of moral scenarios when both have one option where great harm to one person would prevent harm to a greater number.

Perhaps partly due to this mistake, the current literature often seems to confuse two distinct aims of research. The personal/impersonal distinction might be an attempt to:

1. identify the precise psychological factor that *distinguishes* responses to the Footbridge and Trolley dilemmas.

This is best achieved by behavioural methods. Neuroimaging can contribute to another aim, that of:

(2) identifying the subpersonal processes that *underlie* the responses on either side of the Footbridge/Trolley distinction.

This second aim *does not* require achieving the first one. All it requires is that we have enough cases that *clearly* fall on either side of the distinction between Trolley and Footbridge. The scenarios must pit impartially better consequences against ‘impersonal’ or ‘personal’ violations of the duty not to harm. The problem is that Greene's battery of dilemmas fails to meet this condition. To demonstrate this, we conducted a survey of five moral philosophers who independently classified the dilemmas in terms of the type of considerations that could plausibly support each of the two choices (Kahane and Shackel, 2008). The resulting classification revealed that many of the dilemmas involved irrelevant considerations, pitting, for example, self-interest against either impartial consequence or duty.^6^ Only 45% of impersonal dilemmas, and only 48% of personal dilemmas, were classified as pitting consequence against duty as required.

This classification was done at a straightforward, intuitive level. It does not draw on some abstruse or controversial interpretation of the dilemmas. To illustrate this point with just one example, consider just this example of a personal dilemma:

You are a young architect visiting one of your construction sites with your boss. Your boss is a despicable individual who makes everyone around him miserable including you. It occurs to you that if you were to push him off of the building you are inspecting he would fall to his death and everyone would think it was an accident. Is it appropriate for you to push your boss off of the building in order to get him out of your life?

Now it is true that this dilemma is ‘personal’ by Greene's definition. But it is not, in the relevant sense, even a *moral* dilemma. To answer ‘Yes’ is not, under any plausible interpretation, to follow utilitarianism. Greene himself defines utilitarian judgment as ‘judging personal moral violations to be acceptable when they serve *a greater good*’ (Greene *et al.*, 2004, p. 390; our emphasis). Killing a person in order to prevent some unpleasantness to oneself and others is not to choose the better consequence overall. It is, at most, an extreme case of amorally following one's self-interest. And this is just one example out of many.

Let us emphasize that our criticism is not that the personal/impersonal distinction is not a *philosophically* accurate formulation of the deontological distinction between Trolley and Footbridge. Philosophers have not yet agreed on such a formulation.^7^ And our criticism is *not* that the personal/impersonal distinction does not precisely capture the feature that *psychologically* differentially influences responses to Trolley and Footbridge. Greene has emphasized from the start that this distinction is not ‘definitive’ and only a useful ‘first cut' (Greene *et al.*, 2001) and other researchers have suggested that this feature is better captured by the traditional Doctrine of Double Effect or something similar (Borg *et al.*, 2006; Cushman *et al.*, 2006). We accept Greene's claim that this is a plausible preliminary hypothesis.

Our criticism is that this battery of dilemmas includes many dilemmas that are not even in the relevant *domain*—not even remotely plausible candidates for being instances of the distinction which is the explanandum of the research. This list of dilemmas is highly unlikely to capture any natural distinction in moral psychology, let alone to shed much light on the dispute between utilitarians and their opponents.^8^

#### 2.2.2 Psychological Measures

Researchers have sometimes used psychological or behavioural measures to classify the dilemmas. Greene *et al.*, 2004 classified personal dilemmas as either easy or difficult and the difficult personal dilemmas were used to investigate the neural antecedents of ‘utilitarian’ versus non-utilitarian judgement. However, the easy/difficult division was not based on the content of the scenarios. It was rather classified on a *subject by subject* basis to reflect reaction times, so that ‘a dilemma that was easy for one person could be difficult for another’ (Greene *et al.*, 2004, p. 396). We are not provided with a subject by subject classification, so we do not know how exactly the dilemmas were classified. However there is no reason to suppose that the sub-class of difficult personal dilemmas captures those dilemmas that pose genuine utilitarian/non-utilitarian choice—a difference in content that could hardly vary from subject to subject. Indeed, Greene *et al.* report that the Footbridge dilemma was typically rated *easy* (Greene *et al.*, 2004, p. 396). Given that the ‘difficult’ category failed to include even the most paradigmatic dilemma manifesting the distinction Greene set out to explain, there is some cause for doubt that his neuroimaging findings are a basis for explaining the Trolley/Footbridge distinction, let alone of the dispute between utilitarians and deontologists.

Koenigs *et al.*, 2007 report that their normal and brain-damaged subjects (except one normal subject on a single case) unanimously rejected the ‘utilitarian’ answer in 8 of the 21 of Greene's personal dilemmas they used. Koenigs *et al.* call these 8 dilemmas ‘low conflict'. Their claim about a utilitarian bias thus concerned only those personal dilemmas on which there was no such agreement, dilemmas they label ‘high conflict dilemmas'.^9^

Essentially conceding that his personal dilemmas were an inadequate instrument for investigating utilitarian versus nonutilitarian judgement, Greene *et al.*, 2008 has also adopted Koenigs *et al.*'s classification, implying he now believes that high conflict personal dilemmas are an appropriate instrument.

However, this is again no great improvement. In claiming an abnormal utilitarian bias for their VMPC patients, Koenigs *et al.* set aside the fact that most patients didn't judge in line with utilitarianism in low conflict dilemmas, on the grounds that these are dilemmas where one could make a moral decision on the basis of familiar moral convention. But there is a simpler explanation: according to our classification, 5 out of the 8 ‘low conflict' dilemmas involved egoistic-self-interest versus duty and one is consequence-involving-egoistic-self-interest versus duty or consequence. It is thus not surprising that no subject went for the supposedly ‘utilitarian’ response.

The ‘high conflict' subset of personal dilemmas at least doesn't vary by subject as Greene's ‘difficult’ dilemmas did, and, luckily, a somewhat higher proportion of high conflict dilemmas had the relevant content—the Footbridge case at least was classified as ‘high conflict'. However, this distinction again relies on content-neutral psychological measures to categorise dilemmas and, unsurprisingly, it still does not succeed in fixing a relevant common content. Thus one of the classic cases debated between utilitarian and non-utilitarians, the Transplant Case, was classified as *low* conflict. Finally, not all high conflict dilemmas involved a pure conflict between maximization of wellbeing and a duty not to harm.

To summarise, we have argued that Greene's battery of dilemmas and its various subsets used in later research fail to ‘fix’ the relevant person-level distinction. They fail to do so because, rather than each dilemma consistently opposing better consequence and duty not to harm, the reasons for choosing one way or another across the dilemmas are often not of the relevant moral kinds. Scientists can, of course, use ‘utilitarian’ in some technical sense of their own devising that does not correspond to its standard meaning. But such a use would be highly misleading, and is incompatible with many of the empirical, philosophical and normative claims made by Greene and others on the basis of this body of research. It is thus certainly not correct to suggest, as Koenigs *et al.* do in response to our criticism, that classification of utilitarian responses to dilemmas in terms of their content is merely one legitimate approach out of several (Koenigs *et al.*, 2008, p. E5).

There is a simple solution to this methodological problem: choose dilemmas with the right content. The involvement of moral philosophers in developing stimuli obviously helps (see e.g. Borg *et al.*, 2006). It is possible to increase rigour by using the methodology we deployed to assess Greene's battery: letting moral philosophers independently classify scenarios directly in terms of their content. We have found that although there wasn't always complete convergence in classifying dilemma content, there was usually wide agreement. Such ‘judges’ must be instructed not to appeal to the most sophisticated philosophical theories and distinctions, but to focus on more intuitive interpretations of the sort expected to correspond to what lay moralizers may be plausibly expected to respond to, consciously or not. This is simple enough in many cases, but how to do this in a generally satisfactory way is an issue that requires further consideration. A further possible check would be to use a group of non-philosophers to classify scenarios. In this case there is a complication of providing suitable instruction to ensure that they have adequate depth of understanding of the relevant moral concepts without sullying their philosophical innocence.

#### 2.2.3 Intuitiveness

There is one psychological factor that researchers have ignored so far but which *should* have been controlled for. The research in question starts out from a distinction between the Trolley and Footbridge cases, a distinction that even utilitarians admit is intuitive. That is, most people find it strongly intuitive that we should divert the trolley, and that we shouldn't push the stranger. This is a psychological fact. Given that this psychological difference is either part of the explanandum, or at least directly relevant to it, we should ensure that the pattern of stimuli we use shares this intuitive difference. In existing studies this was not done. Indeed Koenigs' (and Greene *et al.*, 2008) ‘high conflict' dilemmas are precisely ones where consensus was low and thereby could include dilemmas where the utilitarian option was *not* especially intuitive or even counterintuitive. This again is not a good basis for explaining the distinction in intuitiveness between choices in Trolley and Footbridge.

We measured the intuitiveness of responses to nine of Greene's personal dilemmas (along with 23 dilemmas of our own) using 18 independent lay judges. We found that different personal dilemmas vary considerably in their degree of intuitiveness. Indeed we found that in a third of these personal dilemmas neither duty nor utility-maximising was intuitive and for one of them the *utility-maximising* option was clearly the intuitive response.^10^ By ignoring this factor, researchers have been overlooking a potentially important underlying psychological phenomenon, with potentially confounding effects on reported results.

### 2.3 Fixing Moral Theory: Using the Right Range of Dilemmas

Greene *et al.* set out from one familiar point of dispute between utilitarians and their opponents. Personal dilemmas were aimed at modelling this dispute, even if many of those personal dilemmas that were not directly drawn from the philosophical literature were defective in the way described above. Suppose, however, that the flaws identified above were removed and the methodology improved in the ways we suggested. For example, in reply to our criticism, Koenigs *et al.* have used our classification scheme to reanalyze their data using only appropriate dilemmas, reporting they still find statistically significant evidence that ‘VMPC patients are abnormally utilitarian in their moral judgement' (2008, p. E6). Does the data about the responses of such patients justify such claims about their general moral outlook? Or suppose that Greene's fMRI experiments were repeated with similar outcomes using a corrected set of dilemmas. Would this warrant Greene's claims about an association between cognition and utilitarian judgement, or the neural source of the dispute between utilitarianism and Kantian ethics?

We are now considering questions, not about these subjects' *reasons* for judging in certain ways in response to particular dilemmas, but about whether their overall pattern of judgment justifies ascribing to them belief in general *moral theories*. We will argue that the answer to these questions is negative. A piece of evidence mentioned in Koenigs' paper already suggests why. The very same VMPC patients who supposedly exhibit an abnormal utilitarian tendency with respect to high-conflict personal dilemmas also exhibit an abnormal pattern of response in the Ultimatum Game.^11^ Normal subjects tend to ‘punish’ unfair offers in this game, even though this fails to maximize both self-interest and overall utility. If VMPC patients are abnormally utilitarian they would punish unfair offers less than normal subjects. But that is precisely the opposite of what is found. VMPC patients are significantly *more* vindictive than normal subjects (Koenigs and Tranel, 2007). Such vindictive responses are plausibly described as guided by norms of retributive justice in response to unfairness. But both fairness and retributive justice are paradigmatic examples of *deontological* considerations. Damage to the VMPC thus appears to make people respond more to overall utility in *one* context, but *less* so in another. So such patients exhibit both an abnormal utilitarian *and* an abnormal deontological tendency! If such tendencies warranted ascription to such patients of broad moral outlooks, we would be forced to ascribe to them contradictory moral theories.

This example illustrates the conceptual and, consequently, empirical mistake of assuming that being generally disposed to maximize wellbeing in Footbridge style dilemmas by itself implies anything like adherence to utilitarianism. This mistake seems to be based on the following reasoning:

1. In philosophical debate, utilitarians typically defend the claim that, in order to maximize utility, we ought to push the stranger in Footbridge, and non-utilitarians typically argue for the opposite claim.
2. Some lay moralizers believe that it is appropriate to push the stranger, and generally tend to choose to maximize utility in similar dilemmas.
3. Therefore, these lay moralizers have a disposition to make utilitarian judgements, and thus by studying the subpersonal basis for their choices, we can shed light on the source and nature of belief in utilitarianism.

The problem is that the criteria for ascribing belief in utilitarianism are far more demanding than is recognized by this argument. As we shall see in the next section, to conform to some moral theory in some contexts is not at all the same as to follow it.

#### 2.3.1 Ascribing Belief in Utilitarianism

Suppose someone judges that it is right to push the stranger to death to prevent a trolley from killing five others. Does this show this person to be a utilitarian? Or can we at least say that he has made a ‘utilitarian judgement'?

Such a person does seem to judge that, at least in this one case, the greater number of lives saved is a weightier moral consideration than the fact that to achieve this aim we need to directly cause a single person's death. And if this is the choice that maximizes overall wellbeing, then it is also true that this person has judged in conformity with utilitarianism. But notice first that it is not asserted by the Footbridge scenario that pushing the stranger will lead to a better overall outcome. Under different empirical assumptions, utilitarianism would rather sanction the *opposite* choice: indeed many utilitarians think that if we succeeded in overcoming our strong natural aversion to killing others, then, over time, this would have very bad consequences. We may with some plausibility assume that most lay moralizers do not interpret the situation in this way, but this assumption could be mistaken.

But let us assume that those who choose to push the stranger do take this to be the choice that would maximize wellbeing, and thereby indeed *conform* to utilitarianism.^12^ That on its own does not mean that they are *utilitarians*. To follow utilitarianism they must also believe that the *only* thing that determines whether an act is right is whether it maximizes wellbeing. And if they do believe that, then we would expect them to take the choice that maximizes wellbeing in *all* of these dilemmas. But so far as we know, no subject in any reported experiment has responded in this way—not even Koenigs's *et al.*'s brain damaged patients.

Could this inconsistency be explained in terms of a gap between competence and performance? It is hard to see what limitations of memory or attention could explain this clear deviation from a rather simple principle.^13^ Perhaps subjects believe that the supposedly ‘utilitarian’ choice in some of these dilemmas doesn't really maximize wellbeing? There is no evidence for that either, and by this logic it is also possible that many supposedly ‘deontological’ choices really aim to maximize wellbeing.

In any case, even if a subject always chose to maximise wellbeing in this type of dilemma, this would not yet be sufficient to ascribe to him belief in utilitarianism, given that this pattern of choice is not sanctioned *only* by utilitarianism. It is also consistent with many *deontological* theories.

Consider first the point that when someone judges that it is permissible to lie to prevent a murder, this hardly indicates that he *rejects* the moral prohibition against lying, only that he thinks this prohibition is *outweighed* or *overridden* in this kind of context. When someone judges that it is morally permissible to push the man to save the five, this indeed might be because he rejects any morally relevant distinction between Footbridge and Trolley. But it might also be because he accepts such a distinction yet believes that in this case it has been outweighed. This may come out if we ask him whether he would still push the one to save only two (as a strict utilitarian should agree to do).

In any case, to reject a deontological distinction between the duty not to harm and the duty to prevent harm is not yet to endorse utilitarianism. A person who rejects this distinction might still accept many *other* deontological constraints. Importantly, he might still accept a distinction between harm and benefit and thus give priority to the duty to prevent harm over beneficence, the duty to benefit. He might refuse, for example, to push an innocent stranger to death in order to make even a whole universe ecstatically happy. And such a person could still believe, for example, that we must never tell lies or break promises, or that we are allowed to give the welfare of members of our family priority over that of strangers.^14^

There is, after all, a considerable overlap between what utilitarianism and many deontological theories require or forbid, given that nearly all deontological theories recognize the moral significance of outcomes, and duties of beneficence will very frequently prescribe the same acts prescribed by utilitarianism. What distinguishes utilitarianism is that it takes the maximization of aggregate wellbeing to be the *only* thing of moral significance, and to govern *all* moral choices. To fail to be a utilitarian, it is enough to accept one principled exception. So to legitimately ascribe anything even approximating belief in utilitarianism, we need to know about subjects' responses to a *far* wider range of dilemmas than that used in present studies.

Isn't it very plausible, however, to expect that someone who was willing to push a stranger to death to save several others would also be willing, for example, to lie to prevent a significant harm? This might sound like a plausible empirical assumption, but the reported behaviour of VMPC patients already makes it doubtful. And it appears to be false: in unpublished research we found *no* significant positive correlation between subjects' answers to these two kinds of dilemma (Kahane *et al.*, submitted). We cannot simply assume that the moral views of lay moralizers add up to a philosophically consistent overarching moral theory.

#### 2.3.2 Ascribing Belief in Deontological Theories

Things are simpler when we turn to deontology. It is sufficient for someone to conclude, after reflection, that maximising utility is not morally required in *some* case to count as a non-utilitarian. So if a subject judges that it is forbidden to push the stranger—and *not* because he believes that *this* is the choice that maximizes wellbeing—then he is not a utilitarian. And to the extent that he consistently conforms to some particular deontological distinction, then we have at least ‘fixed’ one genuine explanandum, whose correlates and antecedents we can now investigate at the sub-personal level—an investigation which might reveal, for example, that this type of deontological judgement is driven by emotion.

The problem with the way the current literature discusses deontology lies elsewhere. There is a tendency in that literature to contrast utilitarianism with Kant's ethics, implying that lay moralizers who believe we mustn't push the stranger in Footbridge are following Kantian ethics. But this would also be a mistaken ascription of moral theory, given that Kantian ethics is a systematic moral theory that can also clash with commonsense morality. To accept some deontological *distinction* hardly shows that one believes in any particular deontological *theory*, let alone in Kantian ethics. Kantian ethics might have its psychological roots in commonsense moral intuitions, but it goes well beyond them in ways that also merit empirical investigation. Perhaps because of this conflation, existing research has entirely ignored the minority of subjects that judge that it is impermissible to divert the trolley in the *Trolley case*—a minority of roughly the same size as that of subjects who judge we should push the stranger in Footbridge (Cushman *et al.*, 2006), and arguably of equal scientific interest.

#### 2.3.3 The Moral Outlook of Lay Moralizers

So what moral outlook should we ascribe to those lay moralizers who tend to choose to maximise wellbeing, not invariably, but in far greater frequency than the rest of the population? It is hard to say what such people believe (and they may believe different things) on the basis of the limited evidence we have. One possibility is that they have an uncommon view about the *threshold* of the duty not to harm—about how easily it can be outweighed by the duty to prevent and minimize harm. This would still be a deontological view. Virtually all deontologists accept such thresholds: many would agree that it is permissible, even required, to kill one innocent person to save a *thousand*.

Even if lay moralizers are strongly disposed to prefer utility maximising acts in various contexts they are unlikely to be utilitarians. Utilitarianism is very distant from commonsense morality and from common moral intuitions. As an explicit theory, it is no more than several hundred years old. Neuroscience and psychology might have a part to play in explaining why some people have come to believe in utilitarianism, but the core of the explanation, to the extent that it goes beyond citing reasons and arguments, is more likely to be historical and sociological.

Bearing all this in mind, it is highly misleading to ascribe to lay moralizers belief in utilitarianism or to speak of them making utilitarian judgements or of having an abnormal utilitarian bias. Ascription of general moral outlook is underdetermined by a subject's responses to a narrow range of dilemmas. We must either use a range of dilemmas broad enough to justify ascription to subjects of a certain moral belief, view or theory, or we should characterize what we are investigating in a way that accurately reflects what the pattern of moral judgement in question really justifies ascribing to the subject. In the case of ‘personal’ moral dilemmas, this would be, not ‘utilitarian judgement' (viz. belief in utilitarianism) but the belief that we should give greater priority to the minimization of aggregate harm over constraints against directly causing harm to particular persons.

The need for a broader range of dilemmas introduces a complication. The objective of investigating the correlate of particular types of moral judgments requires a suitably narrow range of dilemmas in order to engage only the relevant sub-personal processes, whilst the objective of ascribing a general moral outlook requires a wide range of dilemmas. Consequently it will be hard to meet these two objectives using a single instrument. It is instead likely that two separate instruments are required, one for the purpose of the investigation of whichever sub-personal processes underlie some specific moral distinction, such as that between the Trolley and Footbridge cases, and a second specifically aimed at identifying overall moral outlook.

But why can't we just directly ask subjects, as we can ask Peter Singer, whether they are utilitarians, or, at least, what were the general considerations that made them judge as they have? Such an approach was at the heart of Kohlberg's studies of moral development (Kohlberg, 1981). It is, for good reasons, now viewed with suspicion. First, as the work of Haidt, 2001 and Hauser *et al.*, 2007 shows, subjects often do not have introspective access to their reasons and may be left dumbfounded when asked to defend their moral judgements. Second, even when subjects do produce reasons, these often seem to be merely post hoc justifications. Third, even when subjects are *consciously* responding to certain reasons or principles, they may not be able to articulate them accurately. Nevertheless this further source of evidence shouldn't be discounted too quickly. Hauser *et al.* found that many subjects seemed to be able to articulate the moral principle that was guiding their judgement in at least some cases, and provide at least tentative evidence that this awareness played a causal role in generating their judgements. Future research should find ways of drawing on both sources of evidence.

We have argued that, although concise and to some extent intuitive, it will often be extremely misleading to characterise the overall moral outlook of lay moralizers as ‘utilitarian’. Instead, we should characterise it as a tendency to give more or less weight to consequence or duty. So we should rate the moral outlook of lay moralizers on two variables:

*Number of deontological constraints*. How many deontological constraints on the maximization of wellbeing does a person accepts?

*Threshold level for overriding these constraints*. How low does he set the threshold for a given deontological constraint, in relation to the maximization of welfare (or even just the minimization of harm)?

The fewer deontological constraints the person accepts, and the lower the threshold he sets for them, the closer his view is, in logical space, to act utilitarianism. But so long as he accepts some deontological constraint, at whatever threshold, he still holds a deontological view, albeit of an increasingly weaker form.

#### 2.3.4 Why a Coarse-Grained Terminology is not Good Enough

It might seem that in this section we have been criticising a straw man. We have cited neuroscientists who claim to have identified the neural processes underlying utilitarian judgment, or an abnormal utilitarian tendency. But it might be objected that these claims are not intended to impute to subjects belief in *utilitarianism*. After all the term ‘utilitarian’ is sometimes used in a loose way in everyday contexts to refer merely to choosing a better outcome, even against a contrary social convention. Indeed, Greene (2008) argues that for the purposes of empirical research, ‘utilitarian judgement' can be defined ‘functionally’, simply as a moral judgment that endorses some act that would lead to the best immediate consequences.^15^

In this section we have tried to clarify the warrant for ascribing belief in utilitarianism to a subject. We saw that merely identifying some judgment as one that conforms to utilitarianism does not suffice, indeed, is not even sufficient for ascribing the narrower aim of maximising wellbeing in some given context. This is not merely a terminological nicety. If researchers ignore these constraints, they are not really explaining what they present themselves as setting out to explain. The research we have reviewed is explicitly framed in terms of the philosophical dispute over trolley problems, and repeatedly refers to figures such as Bentham, Mill and Kant. It has been claimed to *explain* the historical dispute between utilitarians and deontologists in terms of a tension between two neural subsystems (Greene *et al.*, 2004). Indeed, it has even been deployed in arguments *in favour* of utilitarianism (Greene, 2008; Singer, 2005). The significant empirical gap between these ambitious further claims and what current research could have plausibly shown (even if its methodology was flawless) would be immediately apparent if a more accurate terminology was in place.

It might be replied that this is not really a significant gap. For isn't it likely that there exists some subpersonal mechanism that underlies the capacity to estimate aggregate wellbeing, and isn't it highly plausible that this capacity is engaged when subjects choose in conformity with utilitarianism, and, indeed, when utilitarian philosophers reason to a moral conclusion? If this line of thought is correct, then it would seem that just by studying the subpersonal substrates of this type of judgment in lay moralizers we would be singling out the relevant explananda, whether or not these moralizers would be appropriately described as genuine believers in utilitarianism. And so the loose, coarse-grained use of ‘utilitarian judgements' would be vindicated.

This line of thought might be attractive, but it is mistaken. To begin with, it is no more than an empirical hypothesis that there is such a subpersonal mechanism, and that judgments merely conforming to utilitarianism typically engage it. For example, as we saw, subjects may treat harm and benefit as distinct moral categories, and do so through engagement of distinct subpersonal mechanisms, so that estimation of aggregate *harm* in, say, Footbridge need not engage some general capacity to estimate aggregate wellbeing (i.e. the overall balance of benefit over harm).

Furthermore, even assuming that some single mechanism is typically engaged in such judgments, surely it is also equally engaged in *contrary* judgments. For it is absurd to suppose (as is sometimes implied) that a subject judging that it is wrong to push the stranger in Footbridge has not aggregated the total harm and is responding only to the harm to the stranger.

So in fact, when we study what is distinctive of the neural processes underlying the choice to push the stranger we are not studying the capacity to aggregate wellbeing, but the processes that allow the output of this capacity to *dominate* the subject's all-things-considered moral judgment. And, as we saw earlier, these processes might reflect classical forms of *deontological reasoning*, e.g. deciding whether one duty should be given priority over another, or whether aggregate harm passes a certain threshold and therefore outweighs some deontological constraint.

Indeed, such deontological reasoning might underlie many common decisions merely conforming to utilitarianism, a point utterly obscured when we describe such choices as ‘utilitarian’. Hence, what are reported as the uniquely cognitive processes underlying utilitarian judgment might, for all we know, reflect deontological reasoning. Genuine utilitarian thinking, on the other hand, denies the existence of deontological constraints on wellbeing maximization and therefore does not need to outweigh or override such constraints. For research to establish that subjects reason in such a way, that is, do not even recognise such deontological constraints, it would need to meet the conceptual conditions outlined in this section.^16^

Finally, no doubt a genuinely utilitarian psychology would indeed exercise the capacity to aggregate wellbeing. But so would virtually all sane moral outlooks, as well as various forms of self-interested and partial thinking. What is distinctive of utilitarian thinking is not that it engages such a capacity, but that it does not rely on any *other* capacity. The upshot of this is that a subpersonal explanation of the capacity to aggregate wellbeing does not take us very far if our aim is to explain genuinely utilitarian psychology. What is needed is rather an explanation of why genuine utilitarians don't acknowledge any *other* moral consideration, especially given that it seems likely that most utilitarians share many common deontological intuitions. This point is again obscured by the misleading terminology now in currency.

The research we have examined begins with concepts, distinctions and theories drawn from philosophical discussion in ethics, and aims to shed new light on them by studying lay subjects. We have argued that the difficulty involved in taking the step from philosophical discourse to the ascription of belief to lay subjects has not been sufficiently appreciated. The methodological issues we have raised are relevant not only to this particular strand of research. They also surface in other areas of scientific research into morality. More broadly still, they bear heavily on the project of ‘experimental philosophy’, which similarly gathers evidence of lay subjects' intuitions, only on a broader range of topics such as free will and knowledge, and deploys that evidence to philosophical effect.^17^

## 3. Conclusion

The research we have critically reviewed has already influenced discussion in ethics. Leading moral philosophers cite its results as an established scientific fact and use them as premises in arguments about moral theory. For example, Gibbard has recently remarked that:

It seems clear to us we shouldn't push a person in front of a trolley even to save five people with certainty. We *know now* that making this judgment is the result of emotional centres in the brain overpowering centres that operate in a more or less utilitarian way… (Gibbard and Stroud, 2008, p. 185; our emphasis).

The upshot of this article is that such confidence is misplaced. We have argued that an influential strand of current research into the science of moral judgement suffers from significant methodological flaws. If we are correct, then an immediate consequence is that doubt is cast on many of the reported findings of this research, at least until they are replicated using improved methodology. A further consequence is that the grand normative claims that have been made on their basis (Greene, 2008; Singer, 2005) should also be suspended.

Empirical research into moral judgment holds immense promise. Although our discussion was largely critical of current research, our ultimate aim is not negative but forward-looking: to clarify what aims such research can fruitfully pursue, and to identify constraints on the methodology needed to achieve these aims.

## Footnotes

1 On metaethical views on which moral propositions are reducible to psychological states, there may be a tenuous sense in which the psychology of morality might be an empirical investigation into moral truth. But such views typically reduce moral propositions to claims about our *ideal* psychology, hardly a natural object for scientific inquiry.

2 We shall use ‘utilitarianism’ to refer to a simple form of act utilitarianism, as is standard in the literature we shall be discussing.

3 Some philosophers also believe in the possibility of moral tragedy, where a certain act is both required and forbidden.

4 A further problem is that subjects could understand the question in some dilemmas to concern legal rather than moral permissibility.

5 A terminological warning: Greene's personal/impersonal distinction has no relation to Dennett's distinction between the personal and subpersonal.

6 Of impersonal dilemmas, 45% pit egoistic-self-interest against duty, 9% pose a conflict within the categories rather than across them. Of the personal dilemmas, 19% are egoistic-self-interest versus duty, 24% are consequence-involving-self-interest versus duty and 5% are mixed consequence and desert versus duty. No agreement was reached regarding one of the personal dilemmas. On average, 2.7 philosophers rejected the categorisation of each dilemma as belonging to the needed type. On 10 dilemmas the philosophers were unanimous in rejecting the categorisation. See http://www.practicalethics.ox.ac.uk/judges.pdf

7 Though note that on its own, the personal/impersonal distinction would also apply to the choice of a *murderer* deciding between two ways of killing innocent people.

8 Moore *et al.* 2008 criticise Greene *et al.*'s dilemmas for failing to ‘systematically capture the … psychological distinction between the footbridge and trolley problems.’ Importantly, they failed to replicate Greene *et al.* behaviourally when using an improved list of dilemmas.

9 One problem with this terminology is that this categorisation of dilemmas in behavioural terms is often mistakenly interpreted to mark a difference in moral content (e.g. by Moll and de Oliveira-Souza, 2008: ‘One class contained ‘high conflict' (that is, morally ambiguous) …. A second class contained ‘low-conflict' (that is, morally unambiguous) ….’).

10 See http://www.practicalethics.ox.ac.uk/intuitiveness.pdf

11 In the Ultimatum Game, two subjects are assigned the role of either proposer or receiver. They then bargain over a sum of money. The proposer makes an offer on how to divide it. If the receiver accepts this offer, the players are paid accordingly, but if he rejects it, both players receive nothing. Accepting any positive offer, however small, makes them both better off, and so appears to maximise utility. We set aside the complication (ignored by most of the literature) that strictly speaking, since the amounts are small and so overall utility remains the same irrespective of how the money is distributed between the two subjects and the experimenter, a utilitarian would be indifferent between all outcomes of the Ultimatum Game.

12 Weset aside the difficult question of what would count as good grounds for ascribing to subjects belief in rule utilitarianism or indirect act utilitarianism, given the plausible assumption that very few if any lay moralizers are likely to be familiar with these relatively subtle moral theories.

13 Moore *et al.* 2008 do provide suggestive evidence of a competence/performance gap in certain types of utilitarian choice. Subjects with higher working memory capacity were more likely to choose the utility-maximising option when it involved killing someone who is going to die anyway, and were also more consistent in their overall pattern of choice. But differences in working memory capacity were *not* significantly correlated with subjects' choices in straightforward personal dilemmas.

14 Moore *et al.* 2008 report that subjects were more inclined to choose the utility-maximizing option when it also promoted their self-interest.

15 Greene initially suggests that we define ‘utilitarian’ and ‘deontological’ judgements as referring to the judgments typically made by utilitarian or deontological philosophers, but his ‘functional’ definition does not really meet this condition, given that, as he himself notes, deontological philosophers routinely make what he classifies as ‘utilitarian’ judgements. Greene makes the further claim that empirical inquiry will show that terms such as ‘utilitarian’ refer not to moral theories but *in fact* refer to neurocognitive kinds, a claim we find highly implausible but which we can bracket, given that we are only concerned here with whether his proposed terminology is a helpful framework for scientific inquiry into moral judgment.

16 Notice that Greene's subpersonal talk about cognitive processes that ‘control’ or ‘overcome’ a prepotent affective response is consistent with *both* person-level interpretations. It could reflect that subject's feeling an aversion that he discounts as morally spurious (as a utilitarian should), but might equally reflect the subject's taking it to be a *genuine* moral consideration, though one that, in this context, he judges to be outweighed (a form of deontological reasoning).

17 For example, Sosa, 2007 argues that that the paradigms used by experimental philosophers do not rule out that apparent differences in intuitions are really due to different interpretations of the stimuli or questions asked.
